# Health Care in the Information Society, Vol. 1: From Adventure of Ideas to Anarchy of Transition

**DOI:** 10.5195/jmla.2026.2249

**Published:** 2026-01-01

**Authors:** Muhammad Taufan Umasugi

**Affiliations:** 1 umasugi53@gmail.com, Doctoral Program in the Faculty of Medicine, Public Health & Nursing – Gadjah Mada University, Yogyakarta, Indonesia

*Health Care in the Information Society, Vol. 1: From Adventure of Ideas to Anarchy of Transition* is a sprawling, multifaceted examination of the development of health care systems and of the information revolution more generally. Scalable Innovation: A Guide for Inventors, Entrepreneurs, and Innovation Enthusiasts is a history of technological progress that meditates on progress and how this When you can't quite Unpacking these ideas is the ambitious Scratch book, containing little to no consumer product innovations. It is in essence a meditation on the role of information systems as instrument of reform and object of frustration – more especially, in the chaotic transformations that we are living through in the Information Society.

Ingram follows the trajectory from early medical and social ideas of computing to the current revolution in medical computing which will transform medicine and healthcare for good and ill. The book includes a deep personal story of Ingram's journey in health informatics and the development of technology for health, and of Ingram himself.

This volume, the first of three in a series, lays the groundwork for understanding the historical origins, as well as the current turmoil, of the entire industry, before we move to the more concrete, actionable recommendations in the subsequent volumes.

## 1. Introduction – Connecting for Health

The book begins by considering the essential relationship between health care and information. It's Ingram's approach to understanding that medicine and health of the future will be tied at the hip with digital information systems. He examines how the future of health care rests on forging a seamless connection between professionals, patients and digital technologies. The intro as a quick summary of what you can expect.y history, philosophy, and technical details.

## 2. Knowledge, Language, and Reason – From Ancient Times to the Information Age

Ingram explores the philosophical and historical origins from which contemporary health care systems formed. He links ancient notions of knowledge and reason, as conceptualized at the time of Hippocrates and Galen, to the digital information age. Ingram uses the development of medical language and transition from oral to written knowledge as a point of departure to situate contemporary informational systems in medicine.

## 3. Observation and Measurement – From Cubits to Qubits

A particularly useful chapter explaining how medicine progresses with the ascertainment of measurement and observation in technological history. From the standard measure of cubits to more exacting, digitally-rooted measuring devices (in quantum computing, Qubits), Ingram relates how an elevation of observational skill butts into the art of diagnosis, with the digital era straining these borders ever more.

## 4. Models and Simulations – The Third Arm of Science

This chapter considers how models and simulations are used in contemporary health care. “It helps clinicians make decisions from a traditional perspective, wherein the model outcome can be used in predicting disease” Ingram says, noting that models and simulations increasingly make “a signal contribution” in predicting health outcomes, advancing medical research, and even informing surgery. This chapter demonstrates how digital systems serve as the third arm of science and engineering, with capacities noone could ever have dreamed 20 years ago.

## 5. Information and Engineering – The Interface of Science and Society

The chapter is concerned with the ways in which engineering, and especially information technology, have influenced what gets discovered scientifically as well as how healthcare is delivered. Ingram explores the crosssection of these fields, and how computing power has transformed the health industry, from the development of electronic health records (EHRs) to the automation of basic administrative tasks within hospitals.

## ANALYSIS OF USEFULNESS

Health Care in the Information Society is a significant new book in the prestigious series Studies in Health Technology and Informatics. One of its great virtues is that it interconnects history, philosophy, technology and medicine to give a full picture of the information technology and health care partnership. What distinguishes this from Ingram's article is that it is a more reflective piece, and the personal story and hence their “view from the inside” is one of a mover and shaker in [health informatics over the years.

Ingram's perspective is one meant to urge you to question for yourself what the cacophony of information that is generated, used, translated to everyday healthcare, and considered within medicine does to you and your own practice and provision of care.

## POTENTIAL READERSHIP

This book is intended for a broad and diverse audience:

- Health Informatics Professionals and Researchers: Providers of health systems, digital information systems, and data management-based healthcare services can read about past experiences and future possibilities when learning from the history of these technologies in health care.
- Interdisciplinary scholars who work in technology, medical ethics, sociology and philosophy will be interested in Ingram's broad analysis of the growth of health care and its connection to technology.
- Health Care Policy Makers: Ingram's experiences with facing the positives and negatives of reform will be of interest to those involved in establishing healthcare policy and policy about the role of technology in public health structure.
- General Readers Interested in Technology and Society: For readers who are interested in how society grapples with the intersection of technology and health, this title offers an accessible but deep dive into the future health care.

## CONCLUDING THOUGHTS

David Ingram's *Health Care in the Information Age, Vol. 1* is a must-read for those who wish to comprehend the influence of information technology on the future of health care. It is a broadly balanced assessment of the opportunities and challenges which the digitalization of health introduces. By interlacing anecdotes, history, and bleeding-edge tech, Ingram presents an essential guide to the continuing revolution in health care.

No better resource exists to become informed about the history and philosophy of the health care-IT interface. - The book is however also a cautionary story, reminding us to always complement the wonders of technology with the verocity of the human in care. The reflective nature and cross-disciplinary sweep of the book ensure that it is compelling reading for not only a group that is broader than might be assumed.

